# Impact of community-based integrated mass drug administration on schistosomiasis and soil-transmitted helminth prevalence in Togo

**DOI:** 10.1371/journal.pntd.0006551

**Published:** 2018-08-20

**Authors:** Rachel N. Bronzan, Ameyo M. Dorkenoo, Yao M. Agbo, Wemboo Halatoko, Yao Layibo, Poukpessi Adjeloh, Menssah Teko, Efoe Sossou, Kossi Yakpa, Mawèké Tchalim, Gbati Datagni, Anders Seim, Koffi S. Sognikin

**Affiliations:** 1 Health and Development International, Newburyport, MA, United States of America; 2 National Lymphatic Filariasis Elimination Program, Ministry of Health and Social Protection, Lomé, Togo; 3 Department of Fundamental Science, University of Lomé, Lomé, Togo; 4 National Public Health Laboratory, National Institute of Hygiene, Ministry of Health and Social Protection, Lomé, Togo; 5 National Laboratory of the National Malaria Control Program, Ministry of Health and Social Protection, Lomé, Togo; 6 Laboratory of Tsévié Central Hospital, Ministry of Health and Social Protection, Tsévié, Togo; 7 Laboratory of Parasitology, Sylvanus Olympio University Hospital Center, Lomé, Togo; 8 Health and Development International, Lomé, Togo; 9 Health and Development International, Fjellstrand, Norway; 10 National Program for the Integrated Control of NTDs, Ministry of Health and Social Protection, Lomé, Togo; Swiss Tropical and Public Health Institute, SWITZERLAND

## Abstract

**Background:**

Togo has conducted annual, integrated, community-based mass drug administration (MDA) for soil-transmitted helminths (STH) and schistosomiasis since 2010. Treatment frequency and target populations are determined by disease prevalence, as measured by baseline surveys in 2007 and 2009, and WHO guidelines. Reported programmatic treatment coverage has averaged over 94%. Togo conducted a cross-sectional survey in 2015 to assess the impact of four to five years of MDA on these diseases.

**Methodology/Principal findings:**

In every sub-district in the country outside the capital, the same schools were visited as at baseline and a sample of fifteen children age 6 to 9 years old was drawn. Each child submitted urine and a stool sample. Urine samples were tested by dipstick for the presence of blood as a proxy measure of *Schistosoma haematobium* infection. Stool samples were analyzed by the Kato-Katz method for STH and *Schistosoma mansoni*. At baseline, 17,100 children were enrolled at 1,129 schools in 562 sub-districts; in 2015, 16,890 children were enrolled at the same schools. The overall prevalence of both STH and schistosomiasis declined significantly, from 31.5% to 11.6% for STH and from 23.5% to 5.0% for schistosomiasis (p<0.001 in both instances). Egg counts from both years were available only for hookworm and *S*. *mansoni*; intensity of infection decreased significantly for both infections from 2009 to 2015 (p<0.001 for both infections). In areas with high baseline prevalence, rebound of hookworm infection was noted in children who had not received albendazole in the past 6 months.

**Conclusions/Significance:**

After four to five years of MDA in Togo, the prevalence and intensity of STH and schistosomiasis infection were significantly reduced compared to baseline. Data on STH indicate that stopping MDA in areas with high baseline prevalence may result in significant rebound of infection. Togo’s findings may help refine treatment recommendations for these diseases.

## Author summary

Mass drug administration (MDA) is a key component of programs aimed at controlling soil-transmitted helminths (STH) and schistosomiasis, diseases that disproportionately impact individuals in developing countries and adversely affect physical and cognitive development. The World Health Organization recommends evaluating the impact of mass drug administration on the prevalence of these infections after five years of MDA. We present here a study of the impact of four to five years of MDA on the prevalence and intensity of STH and schistosomiasis infections in school children in Togo. The prevalence and intensity of these infections in 2015 were significantly reduced compared to a baseline survey conducted in 2009. Local baseline prevalence in 2009 was the strongest predictor of infection in 2015. These infections are more prevalent in boys than in girls, and in older versus younger children. We found that in areas with high baseline prevalence of hookworm the risk of rebound of infection is high among children who do not receive bi-annual treatment. This information is important for programs weighing the decision to stop MDA in areas where prevalence has been reduced through treatment. This and other findings from this study may help refine treatment recommendations for these diseases.

## Introduction

Schistosomiasis and soil-transmitted helminths (STH) are parasitic diseases that cause significant morbidity worldwide, particularly in sub-Saharan Africa. STH infections are among the most prevalent infections in the world, with approximately 1.5 billion people infected worldwide [1, 2]. These infections can cause weakness, malaise, anemia, and impaired physical and cognitive development [1]. Schistosomiasis can also cause hematuria, anemia, stunting, and reduced ability to learn, and in its chronic form may lead to urogenital complications with bleeding, fibrosis, kidney damage, and bladder cancer [3]. More than 200 million people worldwide are infected with schistosomiasis, and more than 85% of affected people live in sub-Saharan Africa [3].

A key public health strategy against these infections is morbidity control through mass administration of preventive chemotherapy (PC) for children and high-risk adults. Distribution of PC through mass drug administration (MDA) is a highly cost-effective public health program and integration of MDA for multiple diseases in a single campaign yields additional cost savings [4, 5]. Through the coordinated efforts of partners worldwide, including a commitment by major pharmaceutical companies to donate the needed albendazole, mebendazole, and praziquantel, MDA has been dramatically scaled up worldwide in recent years [6]. In 2015, more than 66 million individuals received PC for schistosomiasis, and more than 711 million for STH [7].

While Togo has adhered closely to the WHO guidelines for management of these diseases, the optimal strategies for PC are continually being researched and effectiveness may depend on local disease epidemiology, community acceptance of MDA and other factors. It is therefore incumbent upon countries to monitor the impact of their programs, both to refine control strategies and to share successes and challenges with the global audience to advance knowledge about control of these diseases. The WHO recommends that countries conduct an evaluation after five years of MDA to assess impact on the prevalence of STH and schistosomiasis, and adjust the treatment distribution plan based on the new prevalence data, if appropriate [8]. Here we report on an evaluation assessing the impact of four (in the south of Togo) to five (in the north) years of MDA on the prevalence and intensity of STH and schistosomiasis infection in school-age children in Togo.

## Methods

### Baseline survey and MDA

The country of Togo has a population of more than 7 million people, all of whom are at risk for STH and schistosomiasis infection [9, 10]. In 2009, the Togo Ministry of Health (MOH) launched its Integrated Program for Neglected Tropical Disease (NTD) Control, initiating integrated MDA for the three PC-targeted NTDs currently endemic to Togo: schistosomiasis, STH, and onchocerciasis. Baseline disease prevalence was determined through two surveys, a pilot survey of integrated disease mapping in Binah district in 2007 and a subsequent national survey of the rest of the country in 2009 (henceforth referred to together as “Baseline”, or simply “2009”, data). Together, the two integrated baseline surveys measured the prevalence of schistosomiasis and STH in school-age children in every sub-district in all 35 districts outside the capital of Lomé.

Since 2010, Togo has implemented integrated, community-based (door-to-door) MDA for schistosomiasis and STH, according to World Health Organization (WHO) guidelines [8]. School-age children (SAC) are treated with albendazole for STH either annually or twice per year, and with praziquantel for schistosomiasis either annually or every other year, based on the district and sub-district prevalence, respectively, of the two diseases [10]. In sub-districts with schistosomiasis prevalence ≥50%, praziquantel is also given to adults. UNICEF conducts deworming of children age 1–4 years at child health days in Togo.

### Study design and sampling

To compare prevalence and intensity of infection at baseline and follow-up, in 2015 we repeated the cross-sectional survey that was employed for the baseline prevalence mapping (conducted in 2007 for Binah district and in 2009 for all other districts outside the capital) [10]. The baseline sampling strategy was a novel approach developed by the Togo MOH and the US Centers for Disease Control and Prevention and was designed to capture the focal nature of schistosomiasis by enrolling smaller numbers of children at more sites than is proposed by WHO. We visited all 632 sub-districts of the 35 districts outside the capital. In Binah district in 2007, three villages with expected high prevalence of schistosomiasis were selected in each sub-district, based on reports of hematuria from village health centers or proximity to water. In all other districts, two such villages were sampled per sub-district. In the absence of any risk factors for schistosomiasis the villages were chosen at random. One government-run primary school or government-assisted denominational school was sampled in each village and fifteen children were enrolled at each school. The methodology and results of these integrated baseline assessments have been previously published [10]. In 2015 we visited the same villages as at baseline and in each village, whenever possible, we sampled children from the same primary school that was surveyed at baseline. For schools that had closed or could not be located, the nearest public school was selected as a replacement.

### Enrollment and sample collection at the school

As during the baseline survey, the day prior to the arrival of the field team the sub-district nurse visited the school and instructed the headmaster to select a sample of 30 children aged 6 to 9 years old from Cours Elémentaire classes I and II (equivalent to first and second grades in the USA). This age group was selected to comply with the WHO recommendation of selecting children in their third year of school, while providing an additional grade to ensure sufficient sample size if class sizes were small [8]. Consent forms were sent home with children who had verbally assented to participate in the survey. The following day, children who presented written parental consent and who could provide both urine and a stool sample were enrolled until 15 children from the school had been recruited. Each school headmaster was asked if there had been any school-based deworming activities in the past twelve months. Global positioning system (GPS) coordinates for each school were recorded.

### Field and laboratory analyses

Immediately upon collection in the field, each urine sample was visually assessed, and results were recorded as clear, turbulent or bloody. The samples were tested immediately at the school by urine dipstick (1-parameter urine reagent strips; LW Scientific, Lawrenceville, GA) for the presence of blood, as a proxy measure for *Schistosoma haematobium* infection, as was done at baseline [11]. The semi-quantitative result was recorded as negative, trace, small, moderate, or large amount of blood in the urine, according to the urine dipstick gradations. Any urine dipstick result, other than “negative”, was considered a positive result for *S*. *haematobium*.

After collection of the field samples, the stool and urine samples were transported to the nearest health center where the field team established a mobile laboratory. All stool samples were analyzed by the Kato-Katz method; one slide per child (as in 2009) was prepared and read by a laboratory technician using standard procedures [11], and number of eggs per gram of stool was calculated for *Schistosoma mansoni*, *Ascaris lumbricoides*, *Trichuris trichiura*, and hookworm. The time from enrollment of the first child in the field to the final Kato-Katz analysis in the mobile laboratory was recorded. In the first school visited in each sub-district (i.e. half the schools), one 10-mL volume of urine from each of the first five enrolled children was filtered through a 13mm diameter, 12-micron polycarbonate filter (Sterlitech Corporation, Kent, WA, USA) and examined by microscopy, and *S*. *haematobium* egg counts per 10 mL of urine were recorded.

### Data analysis

Data were double entered into Microsoft Access (Microsoft Corporation, Bellevue, WA), cleaned, and analyzed in STATA 13.1 (StataCorp, College Station, TX). Prevalence estimates between groups (year, sex) were compared using the chi-squared test and prevalence trends across age groups and time-to-reading of Kato-Katz slides were examined using the Cuzick non-parametric test for trend. Disease prevalence across groups was compared using the t-test for unpaired samples with equal or unequal variance (as appropriate). Mixed effect logistic regression models were developed to examine factors associated with infection. The significance threshold for all statistical tests was set at 0.05. Missing data were excluded from estimates of prevalence or intensity of infection. Maps were created using ArcMap 10.4.1 (Esri, Redlands, CA).

### Ethical considerations

The protocol for this study was approved by Togo’s Bioethics Committee for Health Research (Comité Bioéthique de Recherche en Santé; approval number 029/2014/CBRS du 04 décembre 2014). Consent forms were sent home with children who had verbally assented to participate in the survey. The following day, a subset of children who presented written parental consent forms were enrolled in the study.

## Results

### Study population

The results of the 2009 baseline survey have been previously reported [10]. In short, baseline data were collected from 17,100 children at 1129 schools, from October 28 to December 6, 2009, except for Binah district baseline data, which were collected from March 15 to 28, 2007. Only school-level summary data were available for certain measures in Binah. For the 2015 impact assessment reported on here, from February 15 to March 31, 2015, 16,890 children were enrolled from 1,126 schools in 562 sub-districts across Togo (Table 1). Of the 1,126 schools included in the impact assessment, 1,097 were the identical school surveyed in 2009 or 2007 and 26 were replacements for schools that no longer existed or could not be found. Three schools from the 2009 survey were no longer operating and could not be replaced because there was no school in the vicinity. Table 1 shows the age and sex of children surveyed at baseline and in 2015. For the impact assessment, stool samples could be analyzed for 16,889 children and urine dipstick results were available for 16,783 children.

**Table 1:** Study populations of the baseline and 2015 surveys.

		2009^a^ N = 17100			2015 N = 16890	
	Female	Male	Total^c^	Female	Male	Total^d^
Age^b^	n (%)	n (%)	n (%)	n (%)	n (%)	n (%)
All children	6997 (41.0)	10081 (59.0)	17078	7930 (47.0)	8953 (53.0)	16883
6 years	1249 (17.9)	1590 (15.8)	2839 (16.6)	424 (5.4)	420 (4.7)	844 (5.0)
7 years	1827 (26.1)	2636 (26.1)	4463 (26.1)	1342 (16.9)	1306 (14.6)	2648 (15.7)
8 years	2011 (28.7)	2785 (27.6)	4796 (28.1)	2752 (34.7)	2916 (32.6)	5668 (33.6)
9 years	1727 (24.7)	2875 (28.5)	4602 (26.9)	3411 (43.0)	4312 (48.2)	7723 (45.7)
10 years	183 (2.6)	195 (1.9)	378 (2.2)	—	—	—
Mean age [median]	7.68 [8]	7.75 [8]	7.72 [8]	8.15 [8]	8.24 [8]	8.20 [8]

^a^ Except in Binah district, where baseline data are from the 2007 Binah district pilot study.

^b^ Children were significantly older in 2015 than in 2009 (p<0.001) and in both years boys were significantly older than girls (p<0.001).

^c^ Data on age and/or sex are missing for 22 children in 2009. In Binah district at baseline in 2007, only children age 9 and 10 were recruited.

^d^ Data on age and/or sex are missing for 7 children in 2015.

The median time from enrollment of the first child in the field to examination of the final Kato-Katz slide in the mobile laboratory was 4 hours 10 minutes (mean 4 hours 39 minutes). The time from collection to processing was not recorded. We did not observe a decrease in the prevalence of STH infection as time from stool collection to reading of the Kato-Katz slide increased.

### Treatment coverage

**Fig 1:**
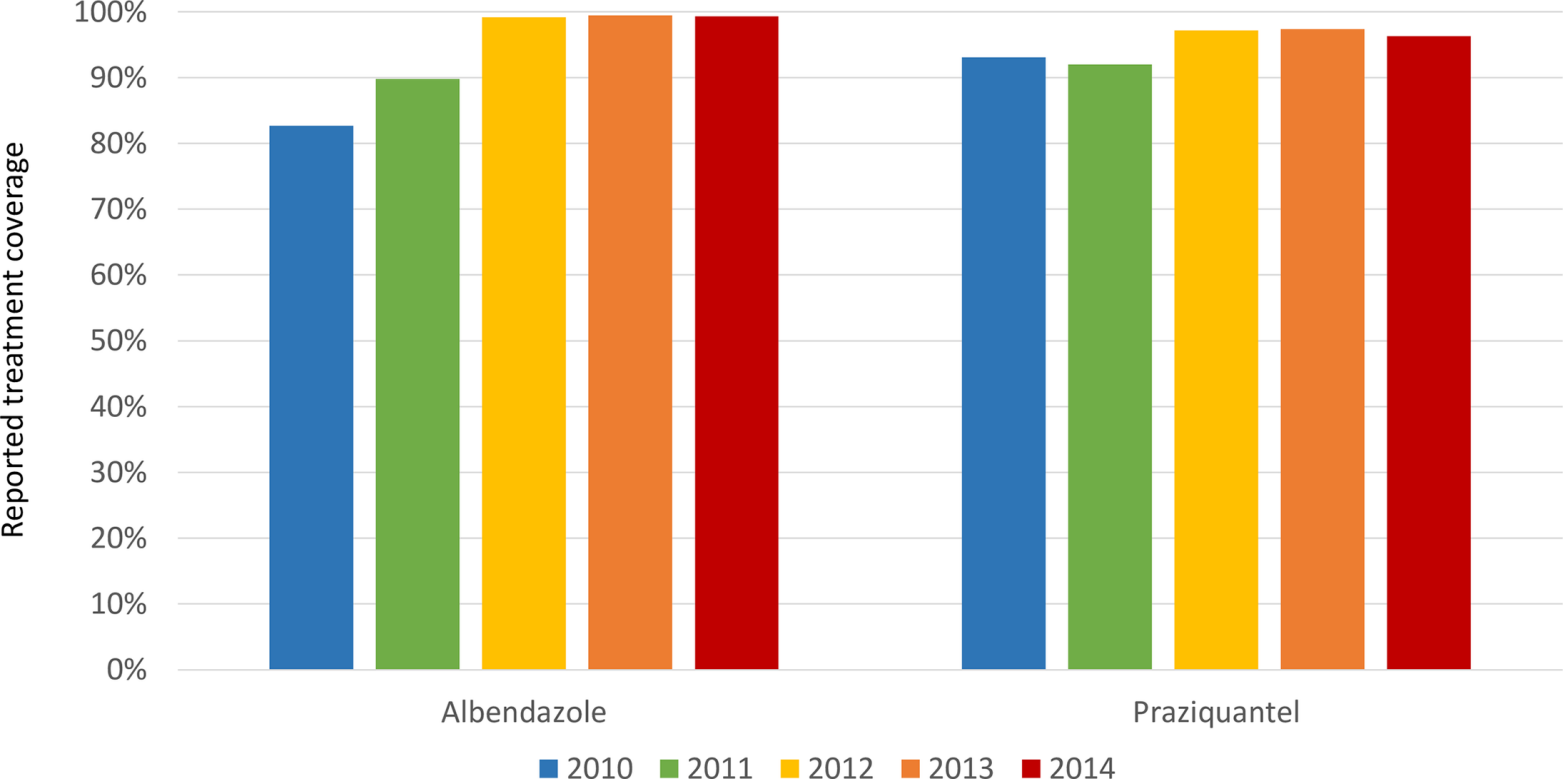
Reported MDA coverage by medication and year.

Fig 1 shows treatment coverage as reported by community drug distributors (number of individuals receiving the medication divided by the number of individuals targeted to receive treatment) by medication and year. No village had poor coverage for more than two consecutive years. We examined 2014 treatment coverage at the village level for those villages in which we had school-based STH and schistosomiasis prevalence estimates and found no correlation between albendazole or praziquantel treatment coverage in 2014 and STH or schistosomiasis prevalence, respectively, in 2015.

### STH

From 2009 to 2015, the overall prevalence of STH decreased significantly from 31.5% in 2009 to 11.6% in 2015 (p<0.001; Table 2). Hookworm was the predominant STH infection at baseline and at follow-up; prevalence of hookworm decreased significantly from 31.0% to 11.1% (p<0.001). Among those with hookworm infection, the egg count per gram of stool was significantly lower in 2015 compared to baseline (p<0.001; Table 2). There were insufficient numbers of *Ascaris* and *Trichuris* infections to examine changes in intensity of infection. At baseline and in 2015, the percent of children testing positive for STH ranged from 0% to 100% across all schools. At baseline, 33 children had mixed infections; in 2015, 29 children had mixed infections.

**Table 2:** Prevalence and intensity of soil-transmitted helminth infections at baseline and in 2015.

		2009^a^								2015							
		Hookworm^b^^,^^h^		*Ascaris*^b^		*Trichuris*^b^		Any STH^b^^,^^i^		Hookworm^c^^,^^g^		*Ascaris*^d^		*Trichuris*^d^		Any STH^e^^,^^g^	
		N = 17097		N = 17097		N = 17097		N = 17097		N = 16887		N = 16888		N = 16888		N = 16886	
Prevalence of infection																	
		n	(%)	N	(%)	n	(%)	n	(%)	N	(%)	n	(%)	n	(%)	n	(%)
		5270	(31.0)	63	(0.4)	41	(0.2)	5342	(31.5)	1868	(11.1)	52	(0.3)	62	(0.4)	1953	(11.6)
Intensity of infection																	
— among all children^f^		N = 16440		N = 16640		N = 16440				N = 16890		N = 16890		N = 16890			
		n (%)		n (%)		n (%)				n (%)		n (%)		n (%)			
	Heavy	133	(0.8)	0	(0.0)	2	(0.0)	—		14	(0.1)	0	(0.0)	6	(0.0)	—	
	Moderate	164	(1.0)	10	(0.1)	7	(0.0)	—		38	(0.2)	9	(0.1)	14	(0.1)	—	
	Light	4915	(29.9)	53	(0.3)	31	(0.2)	—		1816	(10.8)	43	(0.3)	42	(0.3)	—	
	Negative	10878	(68.3)	16374	(99.6)	16397	(99.7)	—		15019	(88.9)	16836	(99.7)	16826	(99.6)	—	
	Missing	3	(0.0)	3	(0.0)	3	(0.0)	—		3	(0.0)	2	(0.0)	2	(0.0)	—	
— among infected children with egg count data		N = 5212		N = 63		N = 40				N = 1868		N = 52		N = 62			
		n (%)		n (%)		n (%)				n (%)		n (%)		n (%)			
	Heavy	133	(2.6)	0	(0.0)	2	(5.0)	—		14	(0.8)	0	(0.0)	6	(9.7)	—	
	Moderate	164	(3.2)	10	(15.9)	7	(17.5)	—		38	(2.0)	9	(17.3)	14	(22.6)	—	
	Light	4915	(94.3)	53	(84.1)	31	(77.5)	—		1816	(97.2)	43	(82.7)	42	(67.7)	—	
	Mean EPG^g^	570		2358		1682		—		289		2027		3220		—	
	Median EPG	168		120		72				96		144		168			
	[range]	[24–36864]		[24–26976]		[24–19896]				[24–9672]		[24–16512]		[24–66432]			

^a^The total number of children surveyed in 2009 is 16440, plus an additional 660 children were surveyed in 33 schools in Binah in 2007, for a total of 17100 children at baseline (“2009”). The 2007 survey in Binah evaluated 20 children per school. The 2009 baseline and 2015 follow-up surveys evaluated 15 children per school. In order to allow a direct comparison of the baseline and follow-up surveys, the data from Binah in 2007 were weighted to represent 15 children per school. Therefore, the prevalence estimates at baseline are not exactly equal to the number of children testing positive divided by the number of children surveyed.

^b^Data on all three species of infection are missing for three children in 2009.

^c^Data on hookworm are missing for three children in 2015.

^d^Data *on Ascaris and Trichuris* are missing for two children in 2015.

^e^STH infection status is missing for four children in 2015.

^f^Data on intensity of infection are not available for the 660 children from Binah district in 2007 (baseline). For hookworm: 1–1,999 eggs per gram of stool (epg) = light infection; 2,000–3,999 epg = moderate infection; ≥4,000 epg = heavy infection. For *Ascaris*: 1–4,999 epg = light infection, 5,000–49,999 epg = moderate infection; ≥50,000 epg = heavy infection. For *Trichuris*: 1–999 epg = light infection; 1,000–9,999epg = moderate infection; ≥10,000 epg = heavy infection.

^g^EPG = Eggs per gram of stool. Mean = arithmetic mean.

^h^Hookworm prevalence and intensity of infection decreased significantly from baseline to 2015, p<0.001 in both instances.

^i^Prevalence of any STH infection decreased significantly from baseline to 2015, p<0.001.

### Schistosomiasis

The prevalence of schistosomiasis decreased significantly from 23.5% in 2009 to 5.0% in 2015 (p<0.001; Table 3). The prevalence of microhematuria, which served as a proxy measure for *S*. *haematobium*, declined from 21.0% at baseline to 4.2% in 2015 (p<0.001). The prevalence of *S*. *mansoni* declined from 3.6% to 0.8% (p<0.001). There were 154 children with mixed infections at baseline and four children with mixed infections at follow-up, but individual-level data were not available for Binah district at baseline so the prevalence of mixed infections could not be assessed for Binah. Among those who were infected, the mean egg count was significantly lower for *S*. *mansoni* in 2015 compared to 2009 (p<0.001, Table 3). In 2015 the majority of *S*. *haematobium* infections were light (Table 3); there was no quantitative measurement of the intensity of *S*. *haematobium* infection in 2009.

**Table 3:** Prevalence and intensity of schistosomiasis infections at baseline and in 2015.

		2009^a^^,^^j^						2015^j^					
		*S*. *haematobium*^b^		*S*. *mansoni*^c^		Any schistosomiasis^d^		*S*. *haematobium*^e^		*S*. *mansoni*^f^		Any schistosomiasis^g^	
		N = 17098		N = 17098		N = 17096		N = 16783		N = 16866		N = 16775	
Prevalence of infection													
		n	(%)	n	(%)	N	(%)	n	(%)	n	(%)	n	(%)
		3638	(21.0)	634	(3.6)	4063	(23.5)	710	(4.2)	129	(0.8)	835	(5.0)
Intensity of infection^h^				N = 17100				N = 2842		N = 16890			
— among all children				n	(%)			n	(%)	n	(%)		
	Heavy	—		50	(0.3)	—		34	(1.2)	11	(0.1)	—	
	Moderate	—		105	(0.6)	—		NA		37	(0.2)	—	
	Light	—		144	(0.8)	—		55	(1.9)	81	(0.5)	—	
	Negative	—		15886	(92.9)	—		2753	(96.9)	16737	(99.1)	—	
	Missing	—		915	(5.4)	—		0	(0.0)	24	(0.1)	—	
— among infected children with egg count data				N = 299				N = 89		N = 129			
	Heavy	—		50	(16.7)	—		34	(38)	11	(8)	—	
	Moderate	—		105	(35.1)	—		NA		37	(29)	—	
	Light	—		144	(48.2)	—		55	(62)	81	(63)	—	
	Mean EPG^i^ or eggs/10mL urine	—		289		—		168		166		—	
	Median [range]	—		120		—		30		72		—	
		—		[24–9672]		—		[1–3720]		[24–2160]		—	

^a^The total number of children surveyed in 2009 is 16440, plus an additional 660 children were surveyed in 33 schools in Binah in 2007, for a total of 17100 children at baseline (“2009”). The 2007 survey in Binah evaluated 20 children per school. The 2009 baseline and 2015 follow-up surveys evaluated 15 children per school. In order to allow a direct comparison of the baseline and follow-up surveys, the 2007 Binah data were weighted to represent 15 children per school. Therefore, the prevalence estimates at baseline are not exactly equal to the number of children testing positive divided by the number of children surveyed.

^b^Data on *S*. *haematobium* are missing for two children in 2009. Data on intensity of infection with *S*. *haematobium* are not available in 2009.

^c^Data on *S*. *mansoni* are missing for two children in 2009. Data on intensity of infection with for *S*. *mansoni* are not available for the 660 children from Binah district and are missing for an additional 255 children in 2009.

^d^Data on *S*. *haematobium* are missing for two children and data on *S*. *mansoni* are missing for two children.

^e^Data on *S*. *haematobium* are missing for 107 children. Data on intensity of infection with *S*. *haematobium* are available for the subset of urine samples that were filtered. Urine filtration was performed on the first five urine samples collected in the first school visited in each sub-district.

^f^Data on *S*. *mansoni* are missing for 24 children.

^g^Data on *S*. *haematobium* and/or *S*. *mansoni* are missing for 115 children.

^h^For *Schistosoma haematobium*: 1–50 eggs/10mL urine = light infection; >50 eggs/10mL urine = heavy infection. For *Schistosoma mansoni*: 1–99 epg = light infection; 100–399 epg = moderate infection; ≥400 epg = heavy infection.

^i^EPG = Eggs per gram of stool (for *S*. *mansoni*). Eggs/10mL urine (for *S*. *haematobium*). Mean = arithmetic mean.

^j^p<0.001 for the difference in prevalence of *S*. *haematobium* and *S*. *mansoni* infection, and for the difference in *S*. *mansoni* intensity of infection, between 2009 and 2015.

### Geographic distribution of disease

**Fig 2:**
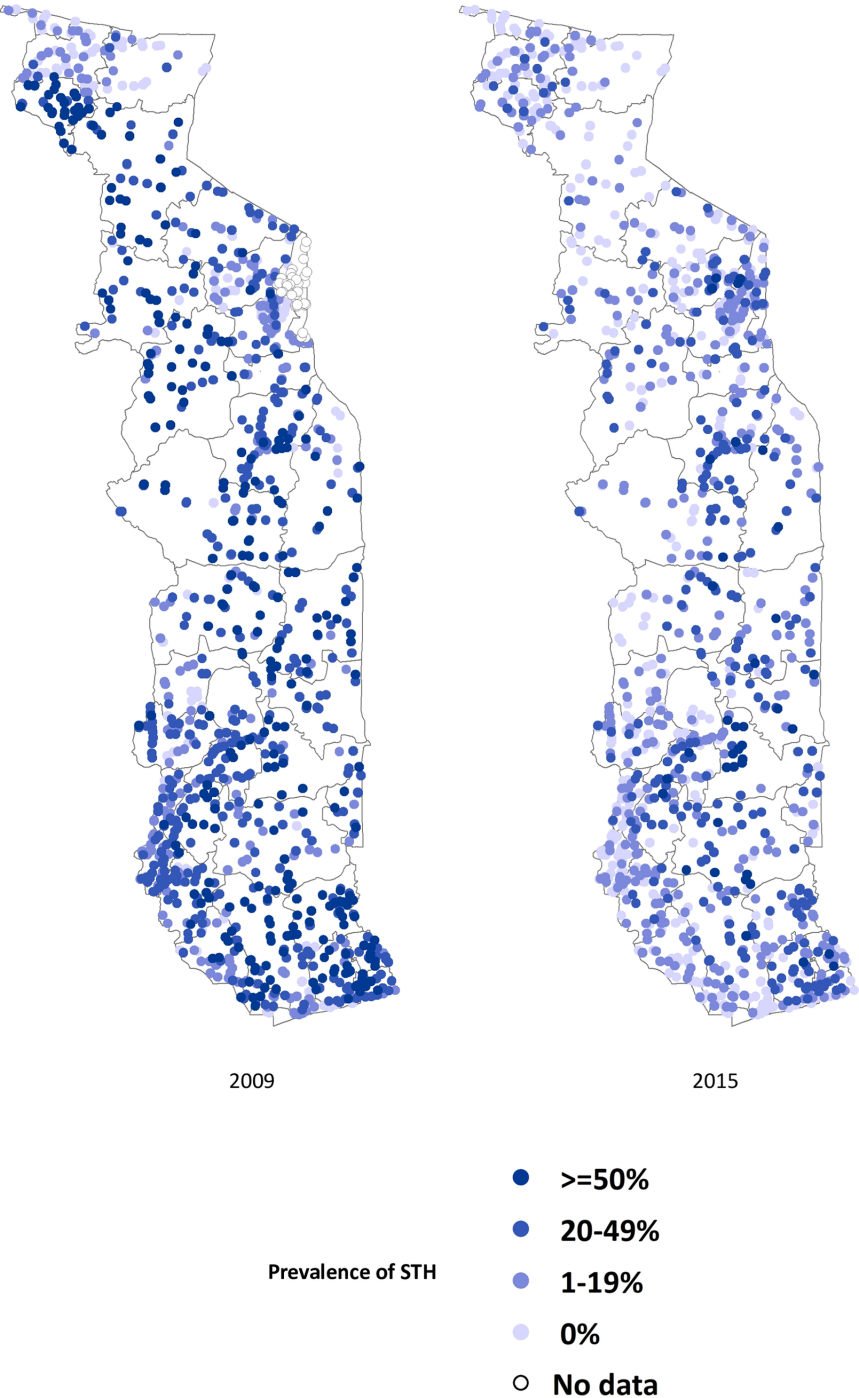
Prevalence of STH in schools surveyed in Togo in 2009 and 2015 (15 children surveyed per school).

**Fig 3:**
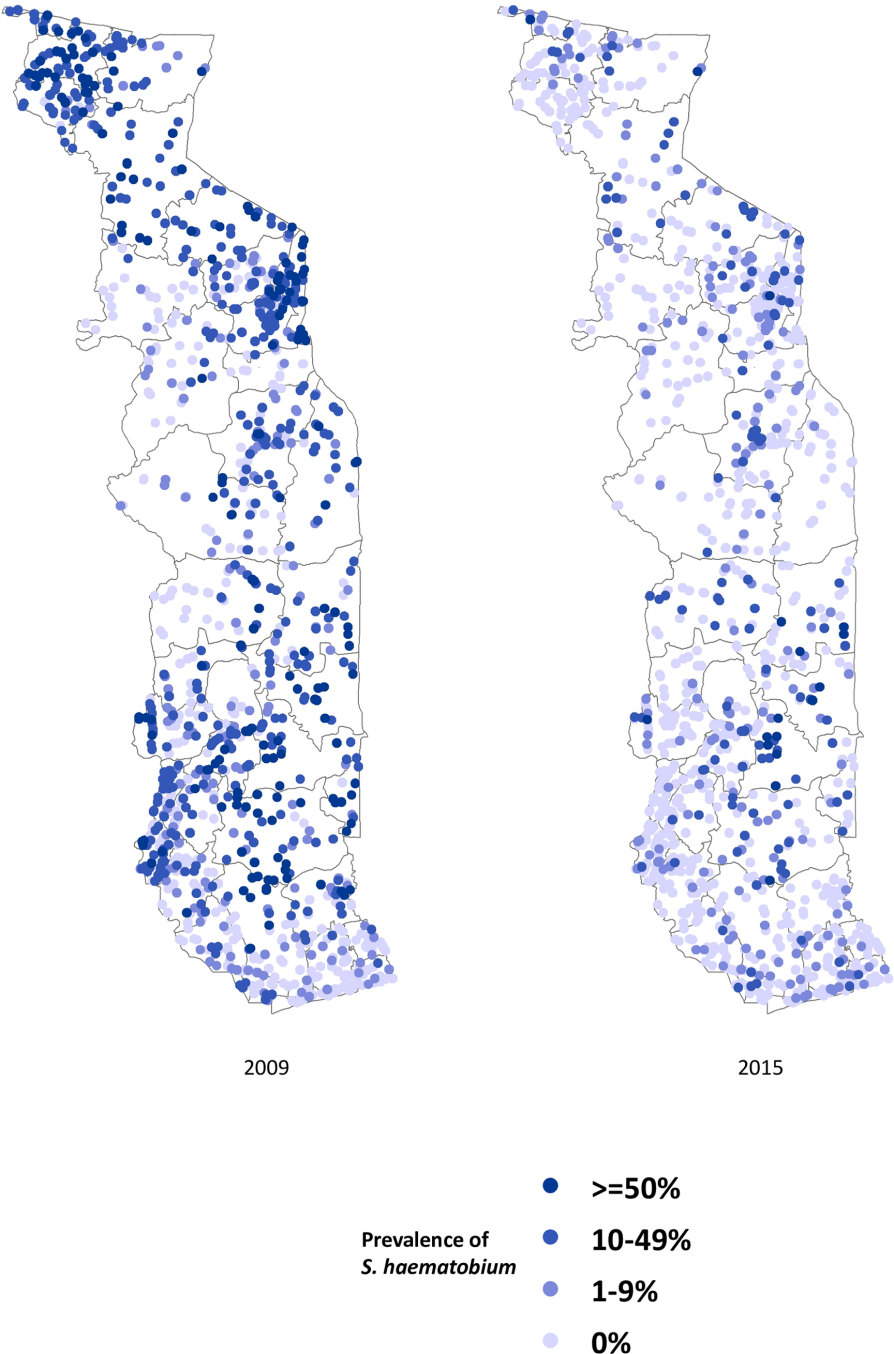
Prevalence of *Schistosoma haematobium* in schools surveyed in Togo in 2009 and 2015 (15 children surveyed per school).

**Fig 4:**
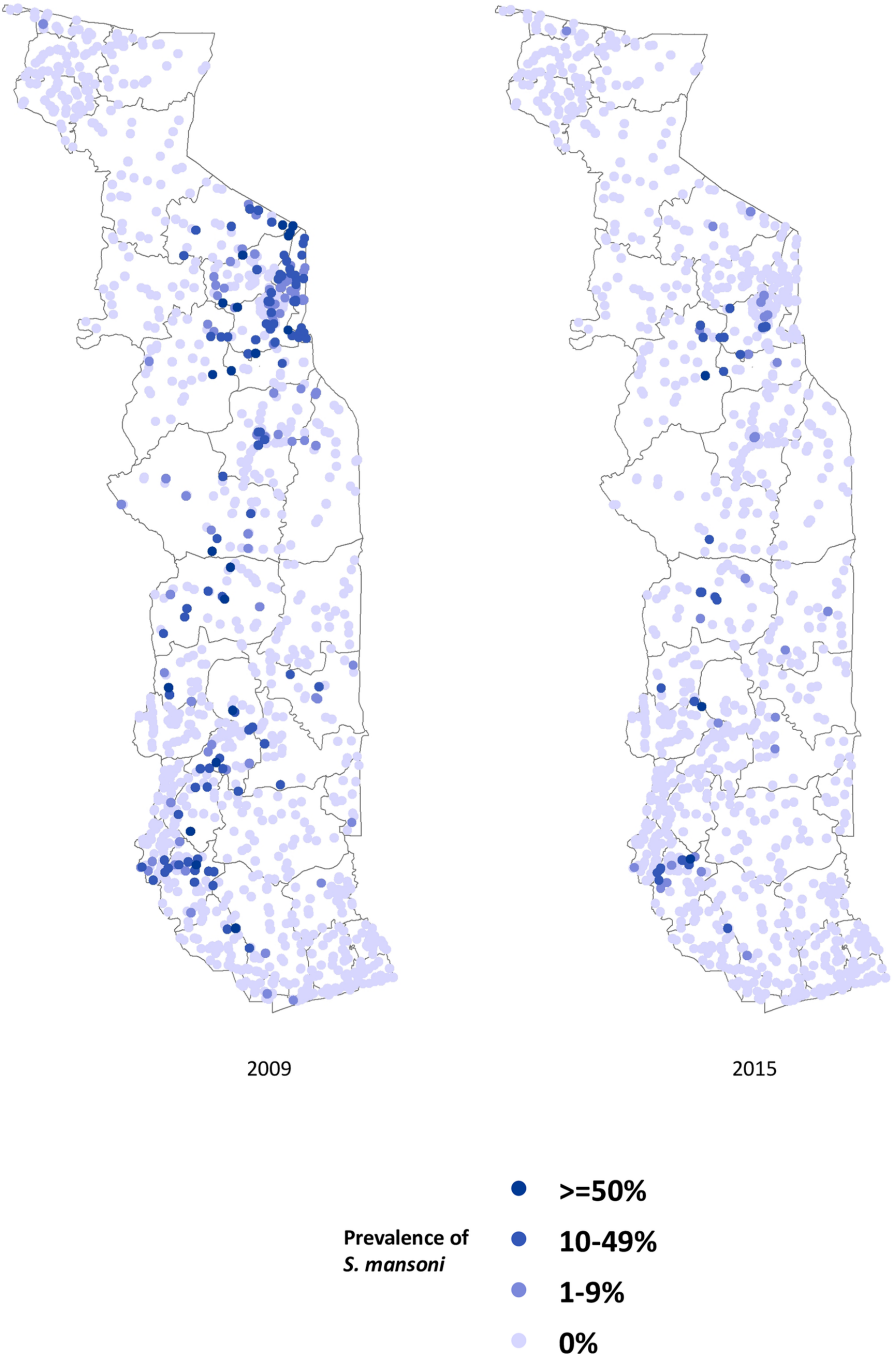
Prevalence of *Schistosoma mansoni* in schools surveyed in Togo in 2009 and 2015 (15 children surveyed per school).

**Fig 5:**
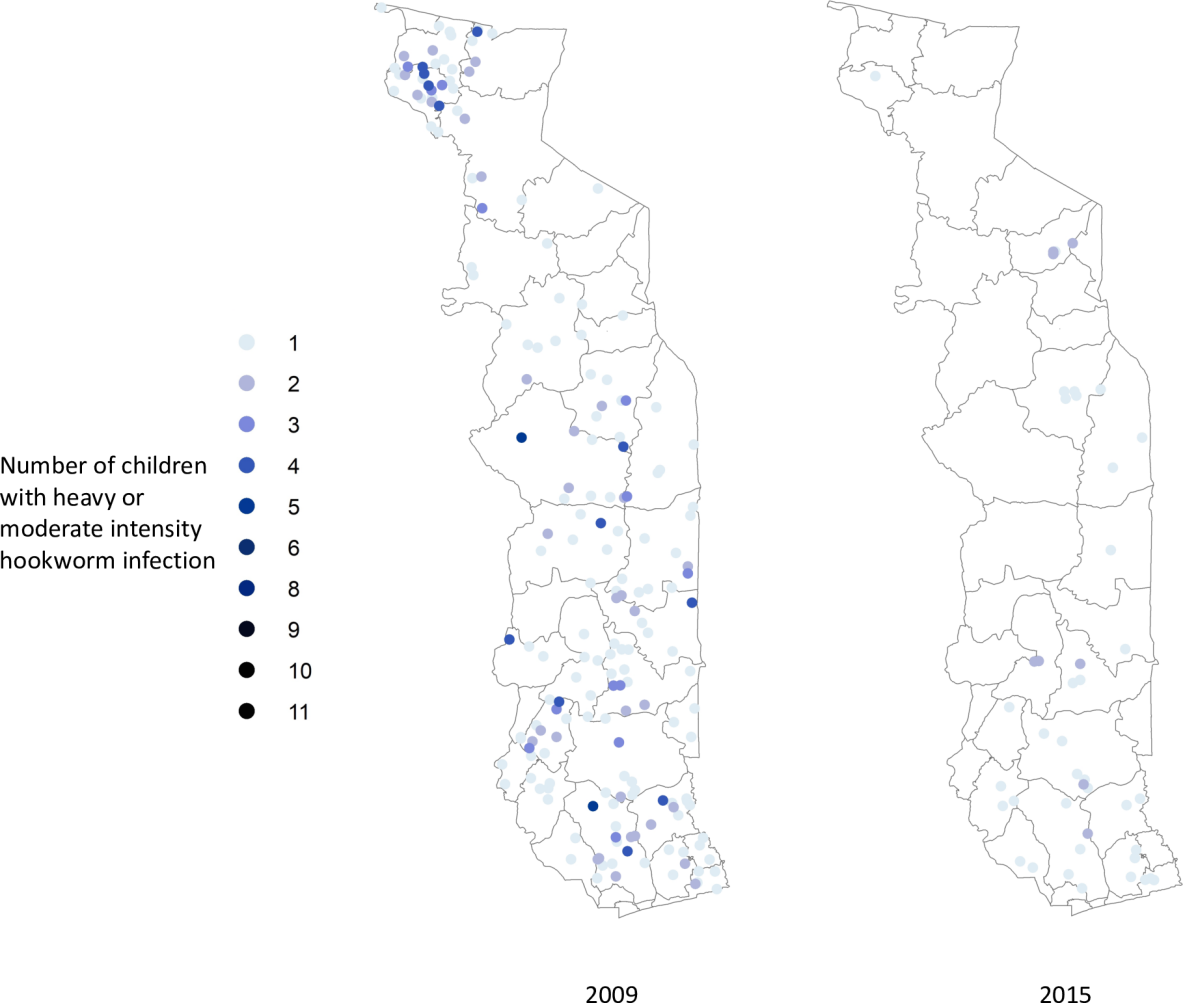
Schools with high- or moderate-intensity hookworm infection in Togo in 2009 and 2015 (15 children surveyed per school).

**Fig 6:**
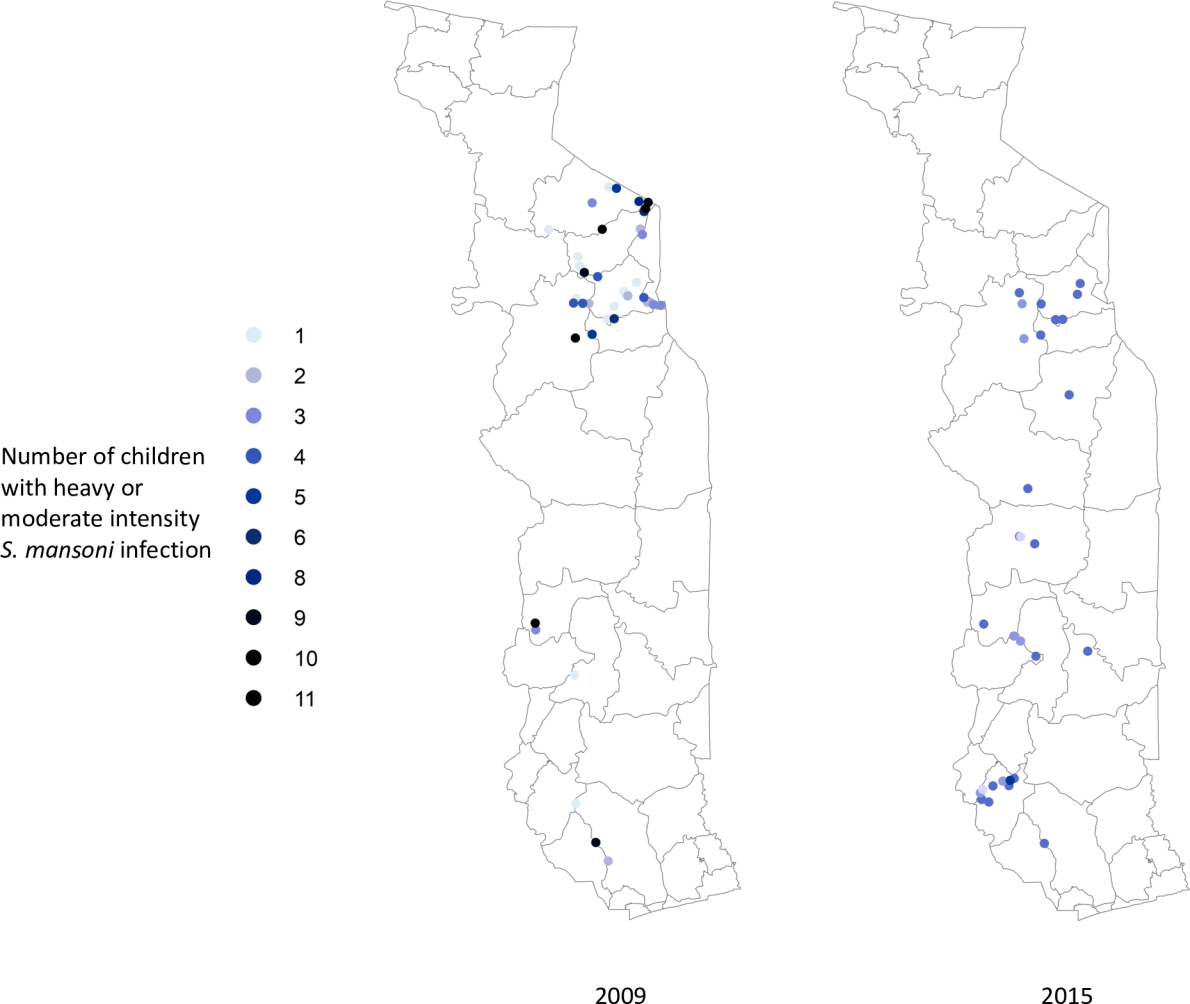
Schools with high- or moderate-intensity *Schistosoma mansoni* infection in Togo in 2009 and 2015 (15 children surveyed per school).

**Fig 7:**
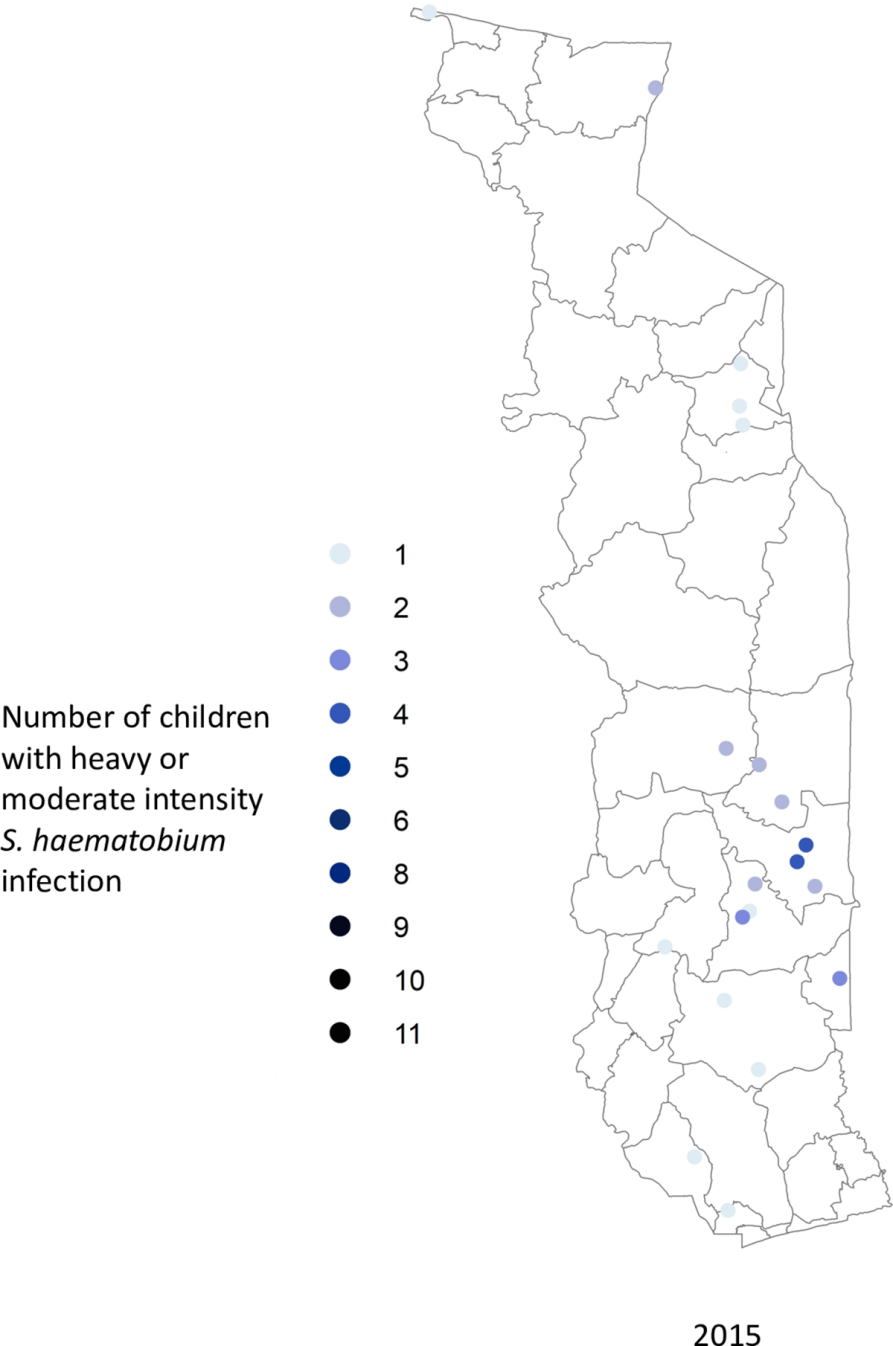
Schools with high- or moderate-intensity *Schistosoma haematobium* infection in Togo in 2015 (15 children surveyed per school).

Figs 2–4 show the prevalence of STH, *S*. *haematobium* and *S*. *mansoni* infection, respectively, at each school in 2009 and 2015 (15 children surveyed per school). Significant reductions in the prevalence of all three diseases are evident in all areas of the country. Figs 5–7 show locations of schools where any child had a high or moderate intensity infection with STH, *S*. *haematobium* or *S*. *mansoni*, respectively. For *S*. *mansoni*, children at schools with higher prevalence of infection were more likely to have high or moderate intensity infections.

### Trends in infection with age

For STH, there is a significant trend toward higher prevalence of hookworm at older ages for both boys and girls, and a trend to higher mean egg counts at older ages for boys, in both 2009 and 2015 (p<0.001 in each instance; Table 4). For schistosomiasis, there is a significant trend toward higher *S*. *mansoni* prevalence and mean egg counts with increasing age for both boys and girls in 2015 (p<0.001 in both instances) but not in 2009. For *S*. *haematobium* there is a trend toward higher prevalence of infection at older ages for both boys and girls in 2009 and in 2015 (p<0.001 in all instances) but no trend in intensity of infection in 2015; no data on intensity of infection are available for *S*. *haematobium* in 2009.

**Table 4:** Prevalence and intensity of hookworm and *S*. *mansoni* infection by age and sex in 2009 and 2015.

		Hookworm											
		2009^a^						2015^b^					
		Girls^i^		Boys^i^		All children		Girls^i^		Boys^i^		All children	
		N = 6664		N = 9751		N = 16415		N = 7932		N = 8955		N = 16887	
Age in years		Prevalence^g^	EPG^c^	Prevalence^g^	EPG^g^	Prevalence^g^	EPG^g^	Prevalence^g^	EPG	Prevalence^g^	EPG^g^	Prevalence^g^	EPG^g^
	6	22.4%	536	26.5%	444	24.7%	480	5.4%	292	9.3%	239	7.4%	259
	7	23.1%	467	29.5%	536	26.9%	511	7.6%	161	10.3%	307	8.9%	244
	8	28.5%	494	37.0%	668	33.4%	605	8.4%	244	12.3%	301	10.4%	279
	9	31.22%	449	44.2%	684	39.4%	616	9.5%	297	15.2%	343	12.7%	327
All ages		26.5%	481	35.3%	617	31.7%	570	8.6%	258	13.3%	323	11.1%	299
		*Schistosoma mansoni*											
		2009^d^						2015^e^					
		Girls^j^		Boys^j^		All children		Girls		Boys		All children	
		N = 6667		N = 9749		N = 16416		N = 7919		N = 8947		N = 16866	
Age in years		Prevalence	EPG	Prevalence	EPG	Prevalence	EPG	Prevalence^g^	EPG^g^	Prevalence^g^	EPG^g^	Prevalence^g^	EPG^g^
	6	2.8%	200	3.8%	222	3.3%	213	0.2%	24	0.2%	24	0.2%	24
	7	2.9%	242	3.5%	264	3.2%	257	0.5%	236	0.7%	84	0.6%	141
	8	2.5%	281	3.7%	194	3.2%	225	0.6%	291	0.4%	102	0.5%	210
	9	3.3%	294	3.8%	525	3.6%	445	0.9%	174	1.2%	150	1.1%	159
All ages		2.9%	260	3.6%	305	3.4%	289	0.7%	213	0.8%	132	0.8%	166
		*Schistosoma haematobium*											
		2009^d^						2015^f^					
		Girls^j^		Boys^j^		All children		Girls		Boys		All children	
		N = 6666		N = 9750		N = 16416		N = 7880		N = 8903		N = 16783	
Age in years		Prevalence^h^	EPG^h^	Prevalence^h^	EPG^h^	Prevalence^h^	EPG^h^	Prevalence^h^	EPG^h^	Prevalence^h^	EPG^h^	Prevalence^h^	EPG^h^
	6	15.6%	—	13.6%	—	14.5%	—	3.5%	433	3.4%	42	3.5%	210
	7	19.5%	—	19.5%	—	19.5%	—	4.6%	108	4.7%	47	4.6%	66
	8	19.9%	—	22.8%	—	21.6%	—	4.6%	94	4.3%	489	4.4%	328
	9	19.9%	—	24.6%	—	22.9%	—	3.9%	66	4.2%	102	4.1%	84
All ages		19.0%	—	20.9%	—	20.3%	—	4.2%	146	4.2%	178	4.2%	165

^a^Data on sex or age are missing for 25 children. Data from Binah district are not included as individual-level data were not available.

^b^Data on infection status are missing for 3 children.

^c^In all instances, EPG = mean number of eggs per gram of stool among those children who are infected. For *S*. *haematobium*, urine filtration and egg counts were performed for a subset of participants (see Table 3).

^d^Data on sex, age or infection status are missing for 24 children. Data from Binah district are not included.

^e^Data on infection status are missing for 24 children.

^f^Data on infection status are missing for 107 children.

^g^P<0.001 for trend with age.

^h^P<0.01 for trend with age.

^i^P<0.001 for prevalence of hookworm infection in girls versus boys, in both 2009 and 2015.

^j^P<0.01 for prevalence of *S*. *mansoni* infection in girls versus boys and for prevalence of *S*. *haematobium* infection in girls versus boys, in 2009 only

### Trends in infection by sex

Boys were more likely to be infected with hookworm and had higher mean egg counts than were girls, in both 2009 and 2015 (p<0.001 in each instance; Table 4), but sex was not related to *Ascaris* or *Trichuris* infection. In 2009, the prevalence of *S*. *mansoni* and *S*. *haematobium* infections was significantly higher in boys than in girls overall (p<0.001), but there was no significant difference in mean egg counts among those who were infected (P = 0.08). There was no significant difference between the sexes in prevalence or intensity of schistosomiasis infection in 2015 (p = 0.55).

### Impact of frequency of treatment on prevalence of infection

Albendazole MDA for hookworm was implemented based on the average district prevalence of hookworm, therefore, within districts with moderate STH prevalence (20–49%), which received annual treatment, there were some schools with high (≥50%) STH prevalence. Conversely, within high STH prevalence districts receiving bi-annual treatment there were some schools with moderate STH prevalence. We were therefore able to compare the effect of annual versus bi-annual treatment on the prevalence of hookworm in schools with high (≥50%) prevalence of hookworm at baseline, and in schools with moderate (20–49%) prevalence of hookworm at baseline (Table 5). Schools with high prevalence of hookworm at baseline that received bi-annual treatment had significantly lower mean prevalence at the time of the 2015 assessment than did high prevalence schools that received only annual treatment (Welch’s t-test, p<0.0001), in spite of the schools that received bi-annual treatment having slightly higher baseline prevalence (Welch’s t-test, p = 0.03). There was also a statistically significant reduction in arithmetic mean hookworm egg count. Annual and bi-annual treatment schedules had similar impact on the prevalence of infection in schools with moderate STH prevalence at baseline; mean prevalence of hookworm in 2015 was not significantly different for Moderate-baseline-prevalence schools that received two versus one round of albendazole (p = 0.55).

**Table 5:** Impact of annual versus bi-annual albendazole treatment on the prevalence of hookworm infection.

	Schools with baseline prevalence of hookworm ≥50%^a^		Schools with baseline prevalence of hookworm 20–49%^b^	
	Schools receiving annual MDA	Schools receiving bi-annual MDA	Schools receiving annual MDA	Schools receiving bi-annual MDA
	N = 195 schools, 2,925 children	N = 82 schools, 1,230 children	N = 365 schools, 5,475 children	N = 41 schools, 615 children
*Baseline– 2009*				
Mean prevalence	65.7%	69.4%	32.1%	34.5%
Median prevalence	60.0%	66.7%	33.3%	33.3%
Range	53.3–100%	53.3–100%	20–46.7%	20–46.7%
*Follow-up– 2015*				
Mean prevalence	21.0%	10.9%	11.1%	9.8%
Median prevalence	20%	6.7%	6.7%	6.7%
Range	0–80%	0–46.7%	0–60%	0–46.7%

^a^For schools with high baseline prevalence of hookworm, those receiving biannual MDA had a significantly higher prevalence of hookworm at baseline (Welch’s t-test, p = 0.03) and a significantly lower prevalence of hookworm in 2015 (Welch’s t-test, p<0.0001) than did those schools receiving annual MDA

^b^For schools with moderate baseline prevalence of hookworm, the 2015 prevalence of hookworm was not significantly different for those schools receiving biannual versus annual MDA (p = 0.55).

Praziquantel MDA for schistosomiasis was implemented at the sub-district level according to the WHO treatment guidelines for the higher of the two school prevalence estimates within that sub-district: annual praziquantel (PZQ) MDA for all persons ≥5 years of age (in high-prevalence subdistricts) or PZQ every other year for school-age children only. As a result, all subdistricts containing a high-baseline-prevalence school received annual PZQ treatment. Moderate-baseline-prevalence schools received PZQ yearly or every other year depending on whether the other school in their particular subdistrict had high-baseline-prevalence or not. Table 6 shows the impact of yearly or biennial (every other year) treatment on the prevalence of both *S*. *haematobium* and *S*. *mansoni*. Among all schools with moderate baseline prevalence of *S*. *mansoni*, there was a significantly lower prevalence of *S*. *mansoni* among those schools receiving annual treatment for persons five years and older versus those receiving treatment every other year for school-age children only (Table 6). When only those schools that were last treated in 2014 were compared, there was no difference in the 2015 prevalence of *S*. *mansoni* between moderate baseline prevalence schools treated annually versus every two years (3.8% vs 2.0%, respectively, p = 0.21). Among moderate baseline prevalence schools receiving PZQ every other year, there was a trend toward higher prevalence of *S*. *mansoni* infection in those schools that were last treated in 2013 versus those schools that were last treated in 2014 (7.7% prevalence of *S*. *mansoni* in 2013 vs. 2.0% in 2014; p = 0.07).

**Table 6:** Impact of praziquantel treatment on prevalence of infection in schools with high or moderate baseline prevalence of *S*. *haematobium* and *S*. *mansoni*.

	*S*. *haematobium*		
	Annual treatment for all people age 5 years and older		Treatment every other year for school-age children only
	Baseline *S*. *haematobium* prevalence ≥50%	Baseline *S*. *haematobium* prevalence 10–49%	Baseline *S*. *haematobium* prevalence 10–49%
	N = 161 schools, 2415 children	N = 72 schools, 1080 children	N = 295 schools, 4425 children
Baseline– 2009			
Mean prevalence	70.3%	26.5%	24.7%
Median prevalence	66.7%	23.3%	20.0%
Range	53.3–100%	13.3–46.7%	13.3–46.7%
Follow-up– 2015			
Mean prevalence	11.3%	4.8%	4.19%
Median prevalence	6.7%	0.0%	0.0%
Range	0–100%	0–6.7%	0–87%
	*S*. *mansoni*^*a*^		
	Annual treatment for all people age 5 years and older		Treatment every other year for school-age children only
	Baseline *S*. *mansoni* prevalence ≥50%	Baseline *S*. *mansoni* prevalence 10–49%	Baseline *S*. *mansoni* prevalence 10–49%
	N = 22 schools, 330 children	N = 30 schools, 450 children	N = 42 schools, 630 children
Baseline– 2009			
Mean prevalence	70.9%	23.8%	21.1%
Median prevalence	76.7%	20.0%	16.7%
Range	53.3–93.3%	13.3%-46.7%	13.3–46.7%
Follow-up– 2015			
Mean prevalence	9.1%	2.0%	4.6%
Median prevalence	0%	0.0%	0.0%
Range	0–60%	0–20%	0–60%

^a^For schools with moderate baseline prevalence of *S*. *mansoni*, those receiving annual MDA for all people age 5 years and older had a significantly lower prevalence of *S*. *mansoni* in 2015 (Welch’s t-test, p<0.0001) than did those schools receiving MDA every other year (Welch’s t-test, p = 0.02).

### Multivariable analysis of contributors to STH and schistosomiasis infection

We used backward stepwise selection to generate mixed effects logistic regression models, including a random intercept for schools, to identify factors associated with hookworm, *S*. *haematobium* and *S*. *mansoni* infection in 2015 (Table 7). Baseline prevalence of infection was the strongest predictor of infection in 2015 for all three parasites. For hookworm, biannual albendazole distribution resulted in half the odds of infection in 2015.

**Table 7:** Multivariate logistic regression showing factors associated with individual infection with hookworm, *S*. *haematobium*, or *S*. *mansoni* in 2015.

	Factor of interest		OR	95% CI	p-value
**Hookworm**					
	2009 prevalence of hookworm at that school				
		0%	Ref		
		1–19%	1.9	1.3–2.8	**0.001**
		20–49%	3.5	2.4–5.1	**<0.001**
		≥50%	7.7	5.2–11.5	**<0.001**
	Age				
		6 years	Ref		
		7 years	1.3	0.9–1.7	0.19
		8 years	1.4	1.0–1.96	**0.03**
		9 years	1.9	1.4–2.5	**<0.001**
	Sex				
		Female	Ref		
		Male	1.7	1.5–1.9	**<0.001**
	Rounds of albendazole MDA per year				
		None	Ref		
		One	1.0	0.8–1.4	0.91
		Two	0.5	0.4–0.8	**0.004**
***Schistosomiasis haematobium***					
	2009 prevalence of *S*. *haematobium* at that school				
		0%	Ref		
		1–9%	1.0	0.6–1.7	1.00
		19–49%	1.8	1.0–3.1	**0.05**
		≥50%	4.5	2.2–9.2	**<0.001**
	Age				
		6 years	Ref		
		7 years	1.5	0.8–3.1	0.19
		8 years	2.0	1.0–4.0	**0.04**
		9 years	2.0	1.0–3.9	**0.04**
	Sex				
		Female	Ref		
		Male	0.9	0.8–1.1	**0.4**
	Treatment frequency and target population				
		No treatment	Ref		
		Treatment every other year of school-age children	1.0	0.6–1.8	0.9
		Annual treatment of all people age five years or older	1.8	0.9–3.5	0.1
***Schistosomiasis mansoni***					
	2009 prevalence of *S*. *mansoni* at that school				
		0%	Ref		
		1–19%	2.8	0.5–15.0	0.23
		20–49%	32.9	9.6–112.5	**<0.001**
		≥50%	392	65.3–2355.1	**<0.001**
	Age				
		6 years	Ref		
		7 years	3.3	0.68.2	0.16
		8 years	2.3	0.4–12.4	0.33
		9 years	6.0	1.1–13.3	**0.03**
	Sex				
		Female	Ref		
		Male	1.2	0.8–1.8	0.16
	Treatment frequency and target population				
		No treatment	Ref		
		Treatment every other year of school-age children	2.2	0.8–6.4	0.15
		Annual treatment of all people age five years or older	0.6	0.1–2.1	0.39

Bold p-values are statistically significant at the α = 0.05 level.

## Discussion

Togo has seen a significant reduction in the prevalence of hookworm and schistosomiasis infection in school-age children after four to five years of door-to-door distribution of albendazole and praziquantel to at-risk populations. The significant reduction in the prevalence of these infections is most likely attributable to the four to five years of carefully implemented MDA that has achieved programmatic coverage averaging 94% for albendazole among SAC and 95% for praziquantel among both SAC and adults [12].

This impact assessment was implemented about nine months after the last nationwide MDA and about four months after the second round of treatment in four high-prevalence STH districts, yielding two groups of schools that differ significantly in terms of frequency of treatment and target populations in their communities, and in the time since the last antihelminth MDA in the community. Baseline prevalence of hookworm was the strongest predictor of post-treatment prevalence, and also modified the effect of biannual treatment on disease reduction. In schools with high (≥50%) hookworm prevalence at baseline, we observed a lower prevalence of infection in 2015 among those children receiving bi-annual albendazole as compared to annual albendazole (Table 5). This could reflect greater reduction in prevalence with repeated rounds of MDA and/or less time for resurgence of infection in areas receiving more frequent treatment; the latter is likely an important factor as communities receiving bi-annual treatment were treated four months prior to the evaluation while those receiving annual treatment had not been treated for nine months, allowing time for rebound of infection [13–15]. Schools with moderate prevalence of hookworm at baseline demonstrated the same reduction in prevalence of infection with either annual or bi-annual treatment. The rebound of infection four to nine months after treatment in areas with high baseline prevalence, even after five years of bi-annual MDA, indicates a pressing need for additional interventions to effect long-lasting reduction in the prevalence of hookworm. For *S*. *mansoni*, among schools with moderate baseline prevalence, we also saw a nearly four-fold higher prevalence of infection among individuals last treated two years prior to the 2015 impact assessment as compared with those treated one year prior to the assessment, suggesting a rebound of infection over time, but the post-MDA prevalence of *S*. *mansoni* was low for both groups and the difference in prevalence was of borderline statistical significance. Rebound of hookworm and schistosomiasis infection has been described elsewhere [14–16]. We observed no significant rebound of infection for *S*. *haematobium* among schools treated every other year compared to schools receiving annual treatment.

Hookworm accounted for 97% of STH infection. *Ascaris* and *Trichuris* infections were very rare, which may reflect the decades of ivermectin distribution for onchocerciasis in most villages in Togo. Hookworm disproportionately affected boys, and prevalence rose steadily with age. These age and sex trends have been reported previously [17, 18], but the increase in hookworm prevalence across only three years of age is striking, particularly among boys. Nine-year-old boys were approximately 1.6 times more likely to be infected with hookworm than were six-year-old boys and were two to three times as likely to be infected as were six-year-old girls.

Hookworm infection is unique among the three STH examined because prevalence of infection increases with age and peaks in adults [17, 18]. Togo’s policy of targeting school-age children for treatment and measuring impact in this same group is aligned with WHO recommendations, and also reflects the extent of treatment that the country could support with available funding. However, adults can serve as an unmeasured reservoir of infection that poses a challenge to elimination of hookworm as a public health problem. There was a significantly lower mean baseline prevalence of hookworm (15.7%) in the eight districts that had received MDA through Togo’s Lymphatic Filariasis Elimination Program–high-coverage MDA with ivermectin and albendazole for all persons age five years and older for six to eight years prior to the baseline survey–compared with those districts that had never received any albendazole (36.7%). But there was much variation in the 2009 prevalence of hookworm in the eight LF districts, hookworm prevalence prior to LF treatment is not known, and many factors contribute to differences in hookworm prevalence at baseline and to the effect of MDA on hookworm prevalence. Indeed, at the 2009 baseline, two of the LF districts had hookworm prevalence of 29% and 30% in spite of prior intensive MDA for LF; after 5 years of MDA with albendazole for SAC, while the first district witnessed a reduction in prevalence to 13%, the latter district still had STH prevalence of 27% in 2015, in spite of evidence of good program coverage. Conversely, two LF districts with STH prevalence of 5% and 7% in 2009 showed no change in prevalence in 2015 in spite of having received no albendazole in the interim period. Without baseline data on STH infection prior to LF MDA it is difficult to draw conclusions about the impact of treating adults on the prevalence as measured in children, but certainly additional interventions will be needed to eliminate hookworm as a public health problem [19, 20].

We did not observe a correlation between treatment coverage rates in 2014 and disease prevalence in 2015 for either STH or schistosomiasis. The absence of a correlation may be due in part to the fact that coverage was overall very high and there were few villages with reported poor treatment coverage (<80% coverage) in which we also had school-based data on disease prevalence; 30 schools were located in villages with coverage <80% for albendazole and another 30 schools were in villages with coverage <80% for praziquantel, so the sample size was small. Unmeasured factors that could have contributed to the observed reduction in disease prevalence must be considered. There is certainly un-programmed deworming that occurs in Togo but could not be measured or accounted for in this analysis. At 45% of the sampled schools the schoolmaster reported school-based deworming had occurred in the previous twelve months. Such deworming is typically led by local or regional non-governmental organizations; there is no government-led or supported school-based deworming program in Togo. We found no association between reported school-based deworming and STH or schistosomiasis infection in our mixed effect models, but other unmeasured deworming activities such as self-treatment could have had an impact. Improvements in water, sanitation and hygiene (WASH) infrastructure or practices could also have had an impact on STH and schistosomiasis prevalence, but we could not track those. The ministry of health did not report any large-scale, government-supported WASH activities during the period from 2009–2015. A related publication using our study data examined the relationship between STH infection and individual and school-based WASH characteristics and practices and found that the associations are complex, underscoring the difficulty of establishing the impact of WASH even when practices are documented [21].

The granular nature of the sampling frame used in both the baseline and 2015 surveys revealed the highly focal nature of both schistosomiasis and STH infections, as reflected in Figs 2–7. Programs that develop MDA targets based on prevalence estimates obtained from a small sample of sentinel sites risk over- or under-treating large segments of the population [10]. Granular epidemiological information is of particular importance for national NTD programs aiming to eliminate these diseases as public health problems, as the need for detailed data about where to target interventions increases as disease prevalence drops. In Togo, the distribution of *S*. *mansoni* is particularly geographically circumscribed and high-intensity *S*. *mansoni* infections show pronounced clustering. Distribution patterns such as these may be driven by determinants such as temperature, elevation and distance to large water bodies; these patterns also highlight the need to identify risk factors that affect transmission on sub-district, village, or even smaller spatial scales [22, 23]. Additional epidemiological work is needed to investigate reasons for remaining hot spots of infection, and these activities, along with granular sampling schemes, are costly [10].

This assessment was conducted in the context of monitoring and evaluation of the Integrated NTD Program in Togo, and certain aspects of the methodology pose limitations for the interpretation of the results. Convenience sampling was used to select the children at each school, and these results may not be representative of the prevalence of infection among all children in Togo. Due to the constraints of implementing such a large national field study, only one stool sample was collected per person. Additionally, the time from collection to final reading of stool samples was longer than desired for hookworm, whose eggs are prone to degradation over time [24]. This may have reduced the sensitivity of the Kato-Katz for detecting hookworm, but the same laboratory set-up and procedures were used in 2009, so for the purposes of comparison across years the processing times are likely similar, although time from stool collection to processing was not recorded in 2009. For the baseline survey in Binah district, schistosomiasis data were recorded only at the school level, so dual schistosomiasis infections could not be identified. We therefore took the higher of the *S*. *haematobium* and *S*. *mansoni* prevalence estimates to represent the overall prevalence of schistosomiasis at each school at baseline, effectively assuming that dual infections occurred whenever both infections were observed in a school; this may have resulted in an underestimation of the proportion of children who had infection with at least one of the two species at baseline, which would in turn result in an underestimate of disease reduction from 2009 to 2015. Another limitation relates to Binah district’s 2007 pilot baseline survey, in which recruited children were 9 or 10 years old, rather than 6 to 9 years old. Given the significant increase in prevalence with age, the enrollment of older children at baseline than at follow-up may have exaggerated the impact of MDA in Binah district.

This cross-sectional impact assessment demonstrates that Togo has made significant progress in the control of STH and schistosomiasis through MDA with albendazole and praziquantel. The findings from this impact assessment have been used to amend target populations and treatment frequency to consolidate gains and intensify efforts in those areas with persistent high prevalence of infection; the optimal treatment algorithm may differ for different settings. More frequent treatment for STH resulted in greater reduction of infection and/or less rebound of infection, but elimination of both STH and schistosomiasis as public health problems does not appear attainable with the current treatment algorithms and WASH interventions in Togo. Interventions such as expansion of STH treatment to adults, greater access to clean water, improved sanitation and hygiene, and snail control are likely necessary to eliminate these diseases as public health problems, and the country risks rebound of these infections if funding for these programs and MDA is withdrawn. The findings from this study may help refine treatment recommendations for sustaining control or attempting elimination of these diseases.

## Supporting information

S1 Checklist STROBE checklist. (DOCX)

## References

[1] WHO. Soil-transmitted helminth infections Fact Sheet. January 2017 [cited 2017 July 27]; Available from: http://www.who.int/mediacentre/factsheets/fs366/en/.

[2] Pullan R.L., et al, Global numbers of infection and disease burden of soil transmitted helminth infections in 2010. Parasit Vectors, 2014 7: p. 37 10.1186/1756-3305-7-37 24447578 PMC3905661

[3] WHO. Schistosomiasis Fact Sheet. January 2017 [cited 2017 July 27]; Available from: http://www.who.int/mediacentre/factsheets/fs115/en/.

[4] Turner H.C., et al, Cost-effectiveness of scaling up mass drug administration for the control of soil-transmitted helminths: a comparison of cost function and constant costs analyses. Lancet Infect Dis, 2016 16(7): p. 838–846. 10.1016/S1473-3099(15)00268-6 26897109

[5] Hotez P.J., Mass drug administration and integrated control for the world's high-prevalence neglected tropical diseases. Clin Pharmacol Ther, 2009 85(6): p. 659–64. 10.1038/clpt.2009.16 19322166

[6] WHO, Crossing the billion. Lymphatic filariasis, onchocerciasis, schistosomiasis, soil-transmitted helminthiases and trachoma: preventive chemotherapy for neglected tropical diseases. 2017: Geneva.

[7] WHO, Schistosomiasis and soil-transmitted helminthiases: number of people treated in 2015. Wkly Epidemiol Rec, 2016 91(49–50): p. 585–95. 27934297

[8] WHO, Helminth control in school age children: a guide for managers of control programmes - 2nd ed. 2011.

[9] Bureau Central du Recensement, D.G.d.l.S.e.d.l.C.N., Togo., Recensement général de la population et de l’Habitat.

[10] Dorkenoo A.M., et al, Nationwide integrated mapping of three neglected tropical diseases in Togo: countrywide implementation of a novel approach. Trop Med Int Health, 2012 17(7): p. 896–903. 10.1111/j.1365-3156.2012.03004.x 22594642

[11] WHO, Action Against Worms. 2008(11).

[12] USAID, NTD Database.

[13] Bethony J., et al, Soil-transmitted helminth infections: ascariasis, trichuriasis, and hookworm. Lancet, 2006 367(9521): p. 1521–32. 10.1016/S0140-6736(06)68653-4 16679166

[14] Jia T.W., et al, Soil-transmitted helminth reinfection after drug treatment: a systematic review and meta-analysis. PLoS Negl Trop Dis, 2012 6(5): p. e1621 10.1371/journal.pntd.0001621 22590656 PMC3348161

[15] Yap P., et al, Rapid re-infection with soil-transmitted helminths after triple-dose albendazole treatment of school-aged children in Yunnan, People's Republic of China. Am J Trop Med Hyg, 2013 89(1): p. 23–31. 10.4269/ajtmh.13-0009 23690551 PMC3748482

[16] Njenga S.M., et al, Once a year school-based deworming with praziquantel and albendazole combination may not be adequate for control of urogenital schistosomiasis and hookworm infection in Matuga District, Kwale County, Kenya. Parasit Vectors, 2014 7: p. 74 10.1186/1756-3305-7-74 24552246 PMC3936945

[17] Hotez P.J., et al, Hookworm infection. N Engl J Med, 2004 351(8): p. 799–807. 10.1056/NEJMra032492 15317893

[18] Forrer A., et al, Risk profiling of hookworm infection and intensity in southern Lao People's Democratic Republic using Bayesian models. PLoS Negl Trop Dis, 2015 9(3): p. e0003486 10.1371/journal.pntd.0003486 25822794 PMC4378892

[19] Strunz E.C., et al, Water, sanitation, hygiene, and soil-transmitted helminth infection: a systematic review and meta-analysis. PLoS Med, 2014 11(3): p. e1001620 10.1371/journal.pmed.1001620 24667810 PMC3965411

[20] Freeman M.C., et al, Integration of water, sanitation, and hygiene for the prevention and control of neglected tropical diseases: a rationale for inter-sectoral collaboration. PLoS Negl Trop Dis, 2013 7(9): p. e2439 10.1371/journal.pntd.0002439 24086781 PMC3784463

[21] Baker J.M., et al, The associations between water and sanitation and hookworm infection using cross-sectional data from Togo's national deworming program. PLoS Negl Trop Dis, 2018 12(3): p. e0006374 10.1371/journal.pntd.0006374 29590120 PMC5902041

[22] Brooker S. and Clements A.C., Spatial heterogeneity of parasite co-infection: Determinants and geostatistical prediction at regional scales. Int J Parasitol, 2009 39(5): p. 591–7. 10.1016/j.ijpara.2008.10.014 19073189 PMC2644303

[23] Chadeka E.A., et al, Spatial distribution and risk factors of Schistosoma haematobium and hookworm infections among schoolchildren in Kwale, Kenya. PLoS Negl Trop Dis, 2017 11(9): p. e0005872 10.1371/journal.pntd.0005872 28863133 PMC5599053

[24] Dacombe R.J., et al, Time delays between patient and laboratory selectively affect accuracy of helminth diagnosis. Trans R Soc Trop Med Hyg, 2007 101(2): p. 140–5. 10.1016/j.trstmh.2006.04.008 16824566

