# Supplementary material for: Impact of community-based integrated mass drug administration on schistosomiasis and soil-transmitted helminth prevalence in Togo
Source: PLoS Negl Trop Dis. 2018 Aug 20;12(8):e0006551. doi: 10.1371/journal.pntd.0006551 (PMC6124778; doi:10.1371/journal.pntd.0006551)
Supplement: S1 Checklist — (DOCX) [file pntd.0006551.s001.docx]

***STROBE Checklist***—Checklist of items that should be included in reports of ***cross-sectional studies***

|  | Item No | References to items recommended to be included in cross-sectional studies |
| --- | --- | --- |
| **Title and abstract** | 1 | (*a*) Pg. 2: Cross-sectional mentioned in abstract |
|  |  | (*b*) Pg. 2: Methodology/Principal Findings section summarizes the design of the study and primary results |
| Introduction | | |
| Background/rationale | 2 | Pg. 4  “Schistosomiasis and soil-transmitted helminths (STH) are parasitic diseases that cause significant morbidity worldwide, particularly in sub-Saharan Africa.”  “A key public health strategy against these infections is morbidity control through mass administration of preventive chemotherapy (PCT) for children and high-risk adults.”  Pg. 5  “The World Health Organization (WHO) recommends that countries conduct an evaluation after five years of MDA to assess impact on the prevalence of STH and schistosomiasis…” |
| Objectives | 3 | Pg. 5  “Here we report on an evaluation assessing the impact of four (in the south of Togo) to five (in the north) years of MDA on the prevalence and intensity of STH and schistosomiasis infection in school-age children in Togo.” |
| Methods | | |
| Study design | 4 | Pg. 6  “To compare prevalence and intensity of infection at baseline and follow-up, in 2015 we repeated the cross-sectional survey that was employed for the baseline prevalence mapping…”  “The baseline sampling strategy… was designed to capture the focal nature of schistosomiasis….  “We visited all 632 sub-districts of the 35 districts outside the capital. In Binah district in 2007, three villages with expected high prevalence of schistosomiasis were selected in each sub-district, based on reports of hematuria from village health centers or proximity to water. In all other districts, two such villages were sampled per sub-district. In the absence of any risk factors for schistosomiasis the villages were chosen at random. One government-run primary school or government-assisted denominational school was sampled in each village and fifteen children were enrolled at each school. In 2015 we visited the same villages as at baseline and in each village, whenever possible, we sampled children from the same primary school that was surveyed at baseline.” |
| Setting | 5 | Pg. 6  “We visited all 632 sub-districts of the 35 districts outside the capital. In Binah district in 2007, three villages… were selected in each sub-district... In all other districts, two such villages were sampled per sub-district… One government-run primary school or government-assisted denominational school was sampled in each village and fifteen children were enrolled at each school. In 2015 we visited the same villages as at baseline and in each village, whenever possible, we sampled children from the same primary school that was surveyed at baseline. |
| Participants | 6 | Pg. 7  “As during the baseline survey, the day prior to the arrival of the field team, the sub-district nurse visited the school and instructed the headmaster to select a sample of 30 children aged 6 to 9 years old from Cours Elémentaire classes I and II (equivalent to first and second grades in the USA). Consent forms were sent home with children who had verbally assented to participate in the survey. The following day, children who presented written parental consent and who could provide both urine and a stool sample were enrolled until 15 children from the school had been recruited.” |
| Variables | 7 | Pg 7-8  “…each urine sample was… tested immediately at the school by urine dipstick… for the presence of blood, as a proxy measure for *Schistosoma haematobium* infection… Any urine dipstick result, other than “negative”, was considered a positive result for *S. haematobium.*” “All stool samples were analyzed by the Kato-Katz method; one slide per child (as in 2009) was prepared and read by a laboratory technician using standard procedures [reference to method published by WHO], and number of eggs per gram of stool was calculated for *Schistosoma mansoni, Ascaris lumbricoides*, *Trichuris trichiura*, and hookworm.” |
| Data sources/ measurement | 8 | Pg 7  “Each urine sample was… tested immediately at the school by urine dipstick… for the presence of blood, as a proxy measure for *Schistosoma haematobium* infection… Any urine dipstick result, other than “negative”, was considered a positive result for *S. haematobium.*” “After collection of the field samples, the stool and urine samples were transported to the nearest health center where the field team established a mobile laboratory. All stool samples were analyzed by the Kato-Katz method; one slide per child (as in 2009) was prepared and read by a laboratory technician using standard procedures [reference to method published by WHO], and number of eggs per gram of stool was calculated for *Schistosoma mansoni, Ascaris lumbricoides*, *Trichuris trichiura*, and hookworm.”  “Each school headmaster was asked if there had been any school-based deworming activities in the past twelve months.” |
| Bias | 9 | Pg. 6  “To compare prevalence and intensity of infection at baseline and follow-up, in 2015 we repeated the cross-sectional survey that was employed for the baseline prevalence mapping...”  “In 2015 we visited the same villages as at baseline and in each village, whenever possible, we sampled children from the same primary school that was surveyed at baseline. For schools that had closed or could not be located, the nearest public school was selected as a replacement.” |
| Study size | 10 | Pg. 6  “We visited all 632 sub-districts of the 35 districts outside the capital.”  “…three villages…[or]…two such villages were sampled per sub-district… One government-run primary school or government-assisted denominational school was sampled in each village.  Pg. 7  “…children who presented written parental consent and who could provide both urine and a stool sample were enrolled until 15 children from the school had been recruited.” |
| Quantitative variables | 11 | Pg. 8  “Prevalence estimates between groups (year, sex) were compared using the chi squared test and prevalence trends across age groups were examined using chi squared test for trend.” |
| Statistical methods | 12 | (a)  Pg. 8  “Prevalence estimates between groups (year, sex) were compared using the chi-squared test and prevalence trends across age groups and time-to-reading of Kato-Katz slides were examined using the Cuzick non-parametric test for trend. Disease prevalence across groups was compared using the t-test for unpaired samples with equal or unequal variance (as appropriate). Mixed effect logistic regression models were developed to examine factors associated with infection.” |
|  |  | (*b*) as above |
|  |  | (*c*) Missing data were excluded from estimates of prevalence or intensity of infection. Pg. 11, 13, 15, see footnotes of tables Tables 2-4. |
|  |  | (*d*) Pg. 22 “We used backward stepwise selection to generate mixed effects logistic regression models, including a random intercept for schools…” |
|  |  | (*e*) not applicable |
| Results | | |
| Participants | 13* | Pg. 9  (a) “In short, baseline data were collected from 17,100 children at 1129 schools, from October 28 to December 6, 2009. The Binah district 2007 baseline data were collected from March 15 to 28, 2007; only school-level summary data were available for certain measures in Binah. For the 2015 impact assessment reported on here, from February 15 to March 31, 2015, 16,890 children were enrolled from 1,126 schools in 562 sub-districts across Togo (Table 1).” |
|  |  | (b) not applicable |
|  |  | (c) not applicable |
| Descriptive data | 14 | (a) Pg. 9, Table 1 |
|  |  | (b) Pg. 11, 13, 15, see footnotes to Tables 2-4 |
| Outcome data | 15 | Pg. 11, 13, 15, 19-21, 23, Tables 2-7, Figures 1-4 |
| Main results | 16 | (*a*) Pg. 11  “From 2009 to 2015, the overall prevalence of STH decreased significantly from 31.5% in 2009 to 11.6% in 2015 (p<0.001; Table 2). Hookworm was the predominant STH infection in both years; prevalence of hookworm decreased significantly from 31.0% to 11.1% (p<0.001). Among those with hookworm infection, the egg burden was significantly lighter in 2015 compared to baseline (p<0.001; Table 2).”  Pg. 13  “The prevalence of schistosomiasis decreased significantly from 23.5% in 2009 to 5.0% in 2015 (p<0.001; Table 3). The prevalence of *S. haematobium,* as measured by urine dipstick for blood, declined from 21.0% at baseline to 4.2% in 2015 (p<0.001) and the prevalence of *S. mansoni* declined from 3.6% to 0.8% (p<0.001).”  “Among those who were infected, the egg burden was significantly lighter for *S. mansoni* in 2015 compared to 2009 (p<0.001, Table 3).” |
|  |  | (*b*) Pg. 12, Table 2, footnote f – categories for intensity of STH infections  Pg. 14, Table 3, footnote h – categories for intensity of schistosomiasis infections |
|  |  | (*c*) not applicable |
| Other analyses | 17 | Pg. 10  “…we found no correlation between albendazole or praziquantel treatment coverage in 2014 and STH or schistosomiasis prevalence, respectively, in 2015.”  Pg. 15  “For STH, there is a significant trend toward higher prevalence of hookworm at older ages for both boys and girls, and a trend to heavier egg burden at older ages for boys, in both 2009 and 2015 (p<0.001 in each instance; Table 4). For schistosomiasis, there is a significant trend toward higher prevalence of both *S. mansoni* prevalence and heavier egg burden with age for both boys and girls in 2015 (p<0.001 in both instances) but not in 2009. For *S. haematobium* there is a trend toward higher prevalence of infection at older ages for both boys and girls in 2009 and in 2015 (p<0.001 in all instances).”  Pg. 19  “Boys were more likely to be infected with hookworm and have greater egg burden than were girls, in both 2009 and 2015.”  “In 2009, the prevalence of *S. mansoni* and *S. haematobium* infections was significantly higher in boys than in girls overall (p<0.001), but there was no significant difference in mean egg burden among those who were infected (P=0.08). There was no significant difference between the sexes in prevalence or burden of schistosomiasis infection in 2015 (p=0.55).”  “Schools with high prevalence of STH at baseline that received bi-annual treatment had significantly lower mean prevalence at the time of the 2015 assessment than did high prevalence schools that received only annual treatment… Annual and bi-annual treatment schedules had similar impact on the prevalence of infection in schools with moderate STH prevalence at baseline.”  Pg. 20  “There was not a significant difference in the 2015 prevalence or intensity of *S. mansoni* for schools that received treatment annually versus every other year…”  Pg. 21  “…among moderate-baseline-prevalence schools receiving PZQ every other year, there was no statistically significant difference in the 2015 prevalence or intensity of *S. mansoni* infection in those schools that were last treated in 2013 versus those schools that were last treated in 2014.”  Pg. 23  “Baseline prevalence of infection was the strongest predictor of infection in 2015 for all three parasites. For hookworm, biannual albendazole distribution resulted in half the odds of infection in 2015.“ |
| Discussion | | |
| Key results | 18 | Pg. 24  “Togo has seen a significant reduction in the prevalence of hookworm and schistosomiasis infection in school-age children over four to five years of door-to-door mass administration of albendazole and praziquantel to at-risk populations. The significant reduction in prevalence of these infections is most likely attributable to the five years of carefully implemented MDA that has achieved programmatic coverage averaging 94.8% for albendazole among SAC and 95% for praziquantel among both SAC and adults.”  Pg. 25  “In schools with high (≥50%) hookworm prevalence at baseline, we observed a lower prevalence of infection in 2015 among those children receiving bi-annual albendazole as compared to annual albendazole (Table 5).” |
| Limitations | 19 | Pg. 27  “Unmeasured factors that could have contributed to the observed reduction in prevalence must be considered. There is certainly un-programmed deworming that occurs in Togo but could not be measured or accounted for in this analysis.”  “Improvements in water, sanitation and hygiene (WASH) infrastructure or practices could also have had an impact on STH and schistosomiasis prevalence, but we could not track those. The ministry of health did not report any large-scale, government-supported WASH activities during the period from 2009-2015.”  Pg. 28-29  “This assessment was conducted in the context of monitoring and evaluation of the Integrated NTD Program in Togo, and certain aspects of the methodology pose limitations for the interpretation of the results. Convenience sampling was used to select the children at each school, and these results may not be truly representativeness of the prevalence of infection among all children in Togo. Due to the constraints of implementing such a large national field study, only one stool sample was collected per person. Additionally, the time from collection to final reading of stool samples was longer than desired for hookworm, whose eggs are prone to degradation over time. This may have reduced the sensitivity of the Kato-Katz for detecting hookworm,… For the baseline survey in Binah district, schistosomiasis data were only recorded at the school level, so dual schistosomiasis infections could not be identified. We therefore took the higher of the haematobium and mansoni prevalence estimates to represent the overall prevalence of schistosomiasis at each school at baseline, effectively assuming that dual infections occurred whenever both infections were observed in a school; this may have resulted in an underestimation of the proportion of children who had infection with at least one of the two species at baseline, but this would in turn result in an underestimate of disease reduction from 2009 to 2015. Another limitation relates to Binah district’s 2007 pilot baseline survey, in which recruited children were 9 or 10 year olds, rather than 6 to 9 year olds. Given the significant increase in prevalence with age, the enrollment of older children at baseline than at follow-up may have exaggerated the impact of MDA in Binah district.” |
| Interpretation | 20 | Pg. 29  “This cross-sectional impact assessment survey demonstrates that Togo has made significant progress in the control of STH and schistosomiasis through MDA with albendazole and praziquantel. The findings from this impact assessment have been used to amend target populations and treatment frequency to consolidate gains and intensify efforts in those areas with persistent high prevalence of infection; the optimal treatment algorithm may differ for different settings. More frequent treatment for STH resulted in greater reduction of infection and/or reduced rates of reinfection, but elimination of both STH and schistosomiasis does not appear attainable with the current treatment algorithms and WASH interventions in Togo. Interventions such as expansion of STH treatment to adults, greater access to clean water, and improved sanitation and hygiene are necessary to eliminate these diseases as public health problems, and the country risks rebound of these infections if funding for these programs and MDA is withdrawn.” |
| Generalizability | 21 | Pg. 28  “Convenience sampling was used to select the children at each school, and these results may not be representative of the prevalence of infection among all children in Togo.” |
